# Status and challenges for molecular solar thermal energy storage system based devices

**DOI:** 10.1039/d1cs00890k

**Published:** 2022-06-21

**Authors:** Zhihang Wang, Helen Hölzel, Kasper Moth-Poulsen

**Affiliations:** Department of Chemistry and Chemical Engineering, Chalmers University of Technology 41296 Gothenburg Sweden zhihang@chalmers.se; Institute of Materials Science of Barcelona, ICMAB-CSIC 08193, Bellaterra Barcelona Spain; Catalan Institution for Research and Advanced Studies ICREA Pg. Lluís Companys 23 Barcelona Spain kasper.moth-poulsen@chalmers.se

## Abstract

Molecular solar thermal energy storage systems (MOST) offer emission-free energy storage where solar power is stored *via* valence isomerization in molecular photoswitches. These photoswitchable molecules can later release the stored energy as heat on-demand. Such systems are emerging in recent years as a vibrant research field that is rapidly transitioning from basic research to applications. Since a major part of the attention is focused on molecular design and engineering, MOST-based device development has not been systematically summarized and introduced to a broad audience. This tutorial review will discuss the most commonly used and developed devices from a chemical engineering point of view. It is expected that future developers of MOST technology could be inspired by the existing devices, keeping in mind the summarized essential practical challenges towards large-scale implementations and more innovative applications.

## Abstract 2

Molecular solar thermal energy storage systems (MOST) can store solar power *via* valence photoisomerization in molecular photoswitches. MOST concept based devices offer emission-free solar energy storage and release on-demand.

## Graphical abstract

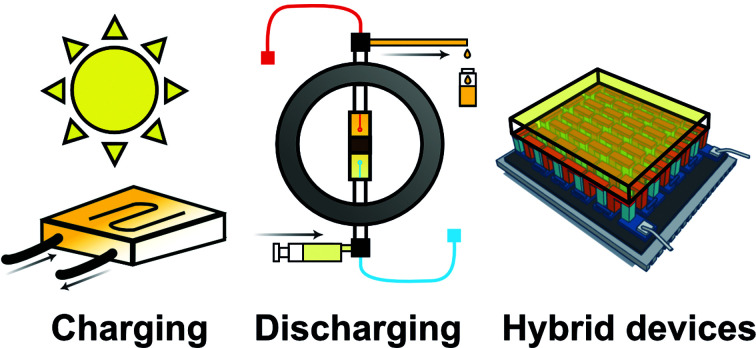

**Key learning points**

1. A brief history of MOST-based devices with a conceptual outline.

2. An introduction to different types of MOST-based devices.

3. An understanding of the current state-of-the-art of MOST-related devices.

4. An understanding of practical issues related to current MOST molecule-based devices.

5. Potential challenges and prospects of making efficient MOST devices.

## 1. Introduction

In the next 30 years, the world population is expected to increase by 25% to 9.9 billion people.^1^ This population increase will come with a continuous increase in energy consumption. Fossil fuels have been the primary energy source over the last decades to cover the energy demand. However, as emissions from the burning of combustible energy sources cause serious environmental effects, such as global warming and air pollution, the latter is also related to health problems,^2^ renewable energy sources, such as wind, geothermal and solar power, are advancing due to their potential as green and sustainable alternatives.^3^

The sun is one of the most abundant energy sources on Earth. The absorption and scattering of incoming photons bring approximately 3.4 × 10^6^ Exajoules of solar energy to the Earth's surface every year. This is 7000–8000 times the annual world primary energy consumption.^4^ Hence, solar energy technologies can potentially meet the growing global energy demands without releasing harmful emissions. However, the use of solar power is still relatively low compared with other energy resources. In 2019, solar power contributed less than 3.6% to the world's total energy production.^3^ One of the limiting factors of solar energy is the intermittency of its production and storage. Hence, more significant implementation of solar power necessitates new scalable technologies for solar energy storage. To provide sufficient and reliable energy, new solar energy storage methods are needed.

Several methods for storing solar energy, such as the use of electrochemical batteries, hydrogen energy storage, and carbon dioxide conversion, are being implemented.^5^ A relatively unexplored method is the use of photoswitchable molecules, called molecular solar thermal energy storage systems (MOST) or solar thermal fuels (STF), which can directly convert and store solar energy as chemical energy. When energy is needed, the high energy photoisomer can release the stored energy on demand in the form of heat. The molecular design of MOST has been reviewed several times in recent years.^6–8^ The main purpose of this review is to describe the current status and challenges of MOST-based devices with regard to energy capture, storage, and release based on different molecular systems.

MOST devices can be divided into three different types: (i) charging, (ii) discharging (heat release), and (iii) hybrid (combining MOST with other energy conversion systems). In the following sections, we will briefly describe the functional principles of the MOST system before introducing the most common and recently developed devices. After this, we will introduce the applications of photoswitchable molecules beyond traditional MOST devices. Finally, we will discuss the challenges of MOST devices and briefly compare them with other solar technologies. We hope this review of existing devices will inspire future MOST technology developers to generate more efficient, practical, real-life applications of solar energy.

## 2. From MOST concept to practical devices

**Fig. 1:**
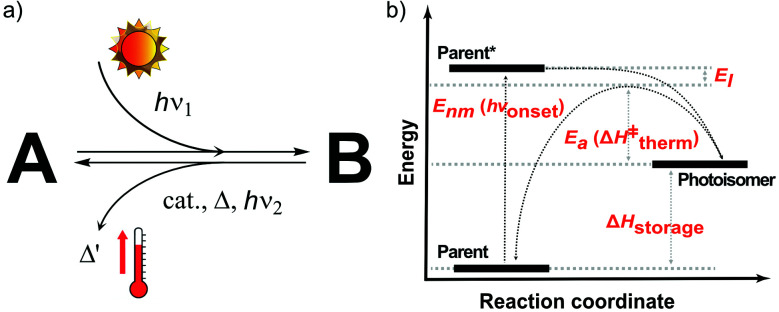
(A) Illustration of a MOST couple. A represents the parent molecule and B is the high-energy metastable photoisomer of A after irradiation (*hν*_1_). The back-conversion from B to A can be realized with an efficient catalyst (cat.), heat (Δ), or light (different wavelength than the conversion process, *hν*_2_). (B) Energy diagram of a MOST system.

The MOST concept originated in 1909, when Weigert suggested photodimerizing anthracene molecules to store solar energy.^9^ In 1979, Xuan *et al.* systematically listed the operation principles of the MOST concept and several criteria that a molecule should fulfill.^10^ This checklist of molecular properties was refined by Yoshida in 1985,^11^ Chernoivanov *et al.* in 1991,^12^ and others.^6,13^ Here, we define organic photoswitchable molecules for MOST as compound A (parent, see Fig. 1A) that can undergo a photon-induced chemical reaction to form photoisomer B (photoisomer). B can then be triggered to switch back to A by light, heat, a catalyst, or another method. In MOST systems, this back-conversion of B to A is associated with the release of stored energy as heat. As shown in Fig. 1B, the charging (or conversion) and discharging (or back-conversion) reactions have different energy levels and processes. The parent state molecule is first excited by a photon with energy *E*_nm_ (or *hν*_onset_) and then photoisomerized to the corresponding high energy metastable photoisomer. Here, Gibbs free energy can be simplified as enthalpies since the activation entropies of most of the isomerization reactions are very low.^13^ Therefore, the energy difference Δ*H*_storage_ between the parent and the photoisomer corresponds to the energy the couple can store. When the stored energy is required, the photoisomer needs a thermal trigger to overcome the activation energy barrier *E*_a_ (or Δ*H*^‡^_therm_). During this charging and discharging process, a part of the energy will be lost due to the thermal relaxation effect (*E*_l_).

Several properties of the MOST systems are strongly correlated, *e.g.* addition of larger substituents to improve *E*_nm_ leads to an increase in the molecular weight, thus lowering the energy storage density. That is why there is still room for improvement in molecular design, even though the concept was conceived as early as 1909.^6^ Yet, it is still very challenging to chemically design an ultimate candidate that can fulfil all criteria of the MOST concept. For instance, by adding additional push-pull units to red-shift the absorption of a parent state molecule, Δ*H*_storage_ can also be lowered.^6^ So far, researchers have focused on improving anthracene, azobenzene, norbornadiene, dihydroazulene, and fulvalene-tetracarbonyl-diruthenium systems.^14^ In addition to optimizing molecular systems, MOST-based devices are also being developed. Since the operating mechanisms of MOST systems are different from those of traditional energy systems, special device concepts need to be developed. In the following sections, we review the device engineering side of the MOST concept, especially how molecular properties can be addressed and linked to the device, and how they affect the performance of the devices.

## 3. MOST charging devices

First, we will consider the theoretical limit of energy storage efficiency of MOST molecules. This efficiency limit implies the maximum efficiency of solar energy storage that is possible for a given MOST molecule. Here, the energy storage efficiency can be calculated as^15–17^1
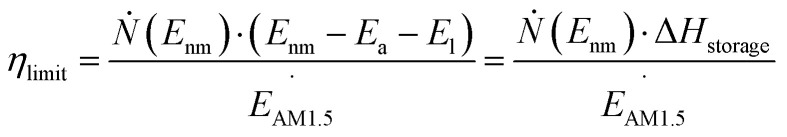
*Ṅ* is the number of absorbed photons at an excitation energy *E*_nm_ per unit time and irradiated area. *E*_a_ is the thermal energy storage barrier as indicated in Fig. 1B. *E*_l_ represents the relaxation energy lost during the photoisomerization process, which corresponds to the energy difference between the excited state and the maximum transition state *via* thermal back-conversion. *E*_AM1.5_ corresponds to the total incoming solar power with an air mass (AM) of 1.5. As an initial approach, it is assumed that all incoming photons from the solar spectrum above a molecular S_0_–S_1_ excitation threshold are absorbed by the parent molecule. Based on eqn (1), it can be estimated that the solar energy storage efficiency can reach up to 12.4% at an S_0_–S_1_ gap of 1.81 eV with a storage time of 24 days at 25 °C.^15^ Later, it was shown that, by combining multiple optimized photoswitches with different onsets of absorption, the efficiency can be increased to ≈21%, where the remaining energy of the solar spectrum is either thermalized during the absorption process or not absorbed by the molecular systems.^16^ This multijunction design has been used for both liquid and solid MOST conversion experiments.^16,18^

In the experimental situation, additional parameters such as concentration, photoisomerization quantum yield, and absorption of photons by the photoisomer must be considered. To do this, eqn (1) can be reformulated as^19^2
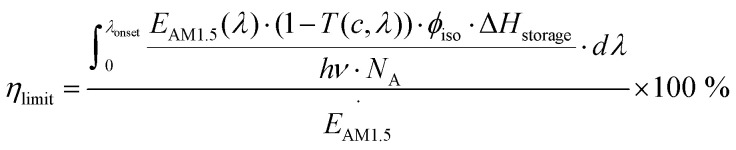
where *T*(*c*, *λ*) is the concentration- and wavelength-dependent transmittance in a specific device with a specific optical path length. *ϕ*_iso_ is the experimental photoisomerization quantum yield of the parent molecule. This equation only focuses on the optical and energy storage properties of the molecular species.

In practice, optical losses, solvent absorption, and thermal back-reaction may lower the efficiency even further.^19^ For instance, from eqn (2), the onset of absorption of the molecule can affect *T*(*c*, *λ*). This is therefore an important limiting parameter of solar energy storage efficiency. The maximum energy storage efficiency of a norbornadiene derivative NBD1 was estimated to be 0.51% with an onset of absorption at 380 nm.^19^ As an example, this efficiency can be improved to 3.8% by applying two NBD units to form NBD2 (see Table 1).^20^ Besides, low *ϕ*_iso_ and/or low Δ*H*_storage_ can also significantly affect the efficiency of the system, and these will be described later when we discuss (fulvalene) diruthenium derivatives and azobenzene derivative tests.

**Table 1:** Typical molecules that have been integrated into devices. The number of the molecular name is ordered by its appearance in the text

Molecules	*λ*_Onset_	*φ*_iso_ (%)	Δ*H*_storage_	*η*_limit_ (%)	*η*_measured_ (%)	Ref.
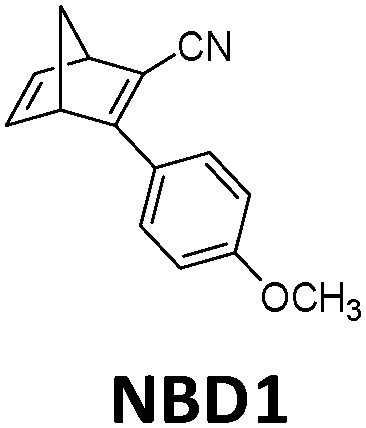	380 nm	61	0.40 MJ kg^−1^ (89 kJ mol^−1^)	0.51	0.03 (4 mM in toluene)	19
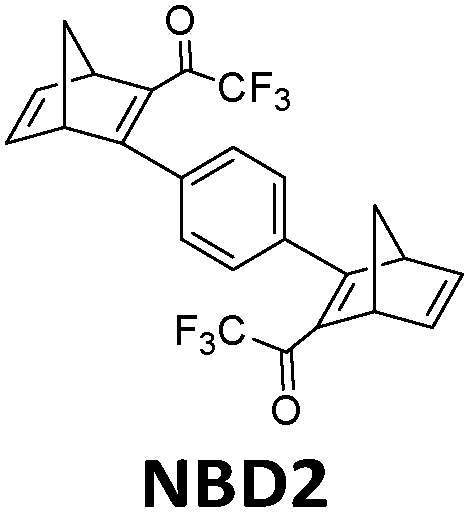	466 nm	77	0.48 MJ kg^−1^ (216 kJ mol^−1^)	3.8	n/a	20
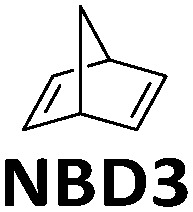	∼300 nm	5	1.0 MJ kg^−1^ (92 kJ mol^−1^)	n/a	n/a	6
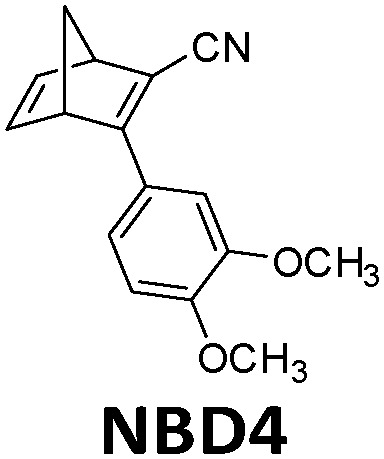	385 nm	68	0.37 MJ kg^−1^ (93 kJ mol^−1^)	0.7	0.5 (0.1 M in toluene)	21
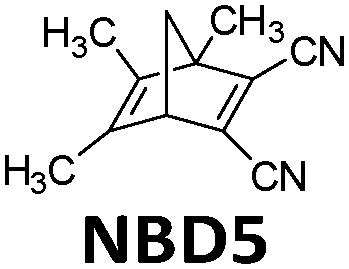	360 nm	96	0.48 MJ kg^−1^ (88 kJ mol^−1^)	n/a	n/a	11
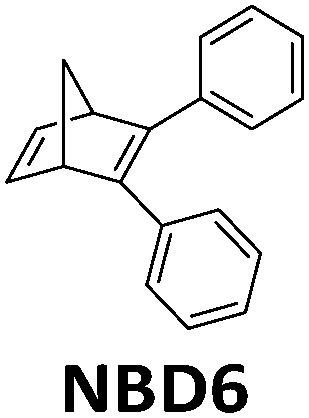	389 nm	60	0.36 MJ kg^−1^ (87 kJ mol^−1^)	n/a	0.1 (0.1 M in toluene)	22,23
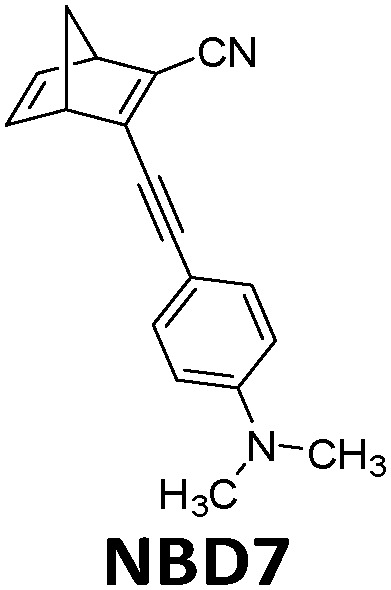	456 nm	28	0.40 MJ kg^−1^ (103 kJ mol^−1^)	n/a	1.1 (0.1 M in toluene)	20
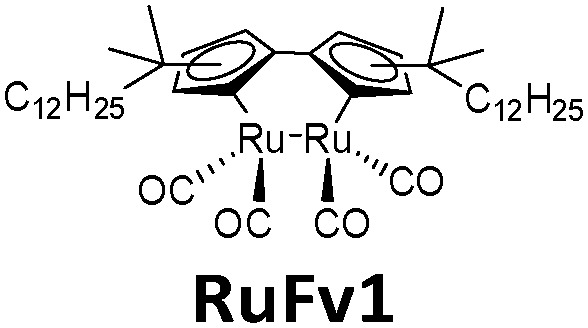	450 nm	0.2	0.11 MJ kg^−1^ (95 kJ mol^−1^)	n/a	0.01 (0.1 M in toluene)	24,25
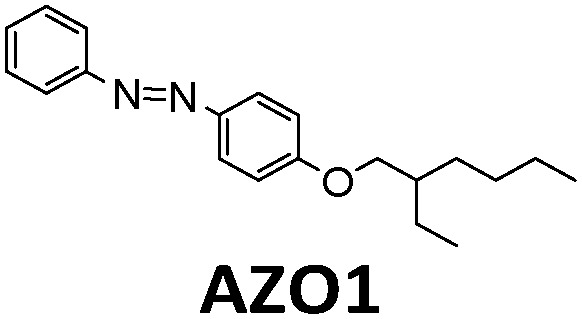	514 nm	21	0.17 J g^−1^ (52 kJ mol^−1^)	0.88	0.009 (5 mM in toluene)	26
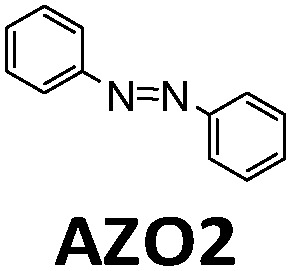	317 nm	44	0.20 MJ kg^−1^ (36 kJ mol^−1^)	n/a	n/a	6,8
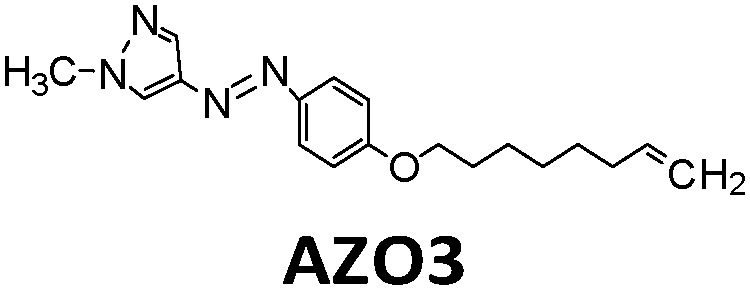	484 nm	41	0.17 MJ kg^−1^ (52 kJ mol^−1^, *cis–trans*), 0.16 MJ kg^−1^ (50 kJ mol^−1^, phase change)	1.2	n/a	27
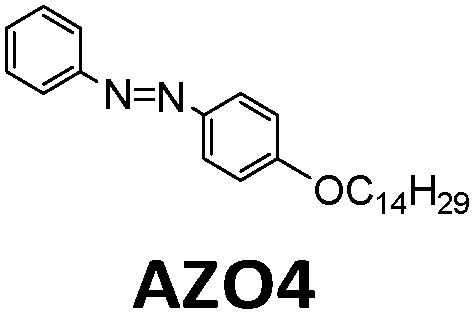	∼400 nm	n/a	0.11 MJ kg^−1^ (44 kJ mol^−1^)	n/a	n/a	28,29
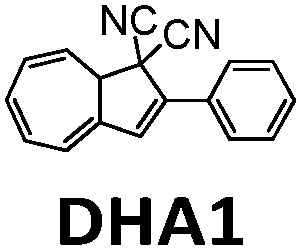	450 nm	60	0.11^a^ MJ kg^−1^ (28 kJ mol^−1^)	0.61	0.1 (4 mM in toluene)	30

a Theoretical value.

The MOST system has a relatively free form-factor in a liquid state, and it can be conveniently transported *via* pumping.^15^ To determine the experimental solar thermal energy storage efficiency for fluidic MOST, the following equation can be used:^23^3
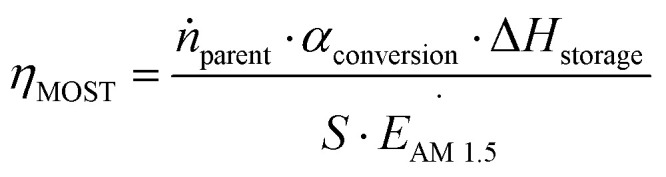
where *ṅ*_parent_ represents the flow speed of the parent molecule in mol s^−1^ and *S* is the effective irradiated area of the device in m^2^. *α*_conversion_ corresponds to the conversion ratio from parent state to photoisomer. This ratio can be calculated using Lambert–Beer's law when the absorptivity of the photoisomer is negligible compared with that of the parent state. If this is not the case, the ratio can also be calculated with the isosbestic point:4
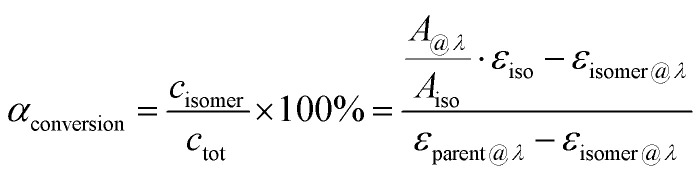
where *c*_isomer_ and *c*_tot_ are the concentrations of the photoisomer and the whole MOST solution, respectively; *A*_*@λ*_ is the experimental absorbance at a certain wavelength *λ*; *A*_iso_ represents the experimental absorbance at the isosbestic point; *ε*_iso_, *ε*_isomer@λ_, and *ε*_parent@λ_ are absorptivities of the specific isosbestic point, photoisomer at *λ*, and parent at *λ*. A major practical challenge of current MOST molecules is that the absorption spectrum of the parent state overlaps with that of the photoisomer and this can significantly affect device performance. From eqn (3), it can be seen that the experimental solar thermal storage efficiency *η*_MOST_ can vary by the conversion ratio *α*_conversion_. This factor can be interpreted as the difference between the absorption spectrum of the parent molecule and that of its corresponding photoisomer. When the spectra largely overlap, an absorption competition can occur during conversion, increasing the time it takes for the solution to be fully charged. As a result, *η*_MOST_ will drop during charging. This intrinsic problem generates an “inner filter effect”. Since ideal zero-spectrum overlap molecules have not been identified yet, low concentrations for practical tests are the most convenient way to demonstrate the basic function.

**Fig. 2:**
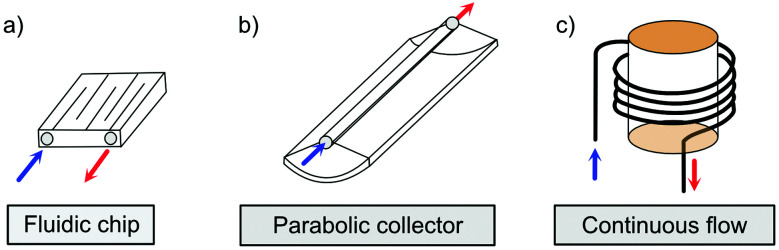
Three types of MOST charging devices. (a) The fluidic chip solar collector contains fluidic channels. By installing the device under solar irradiation, the MOST solution can thus be converted with an appropriate residence time inside the collector (concentrating capacity: ≤1 sun).^24^ (b) The parabolic collector can focus sunlight onto the central tubing with flowing MOST solution (concentrating capacity: 5 to 30 sun).^22^ (c) The continuous flow system uses a central irradiating source with flow tubing surrounding. The concentrating capacity can be varied with different exposure times.^21^

**Fig. 3:**
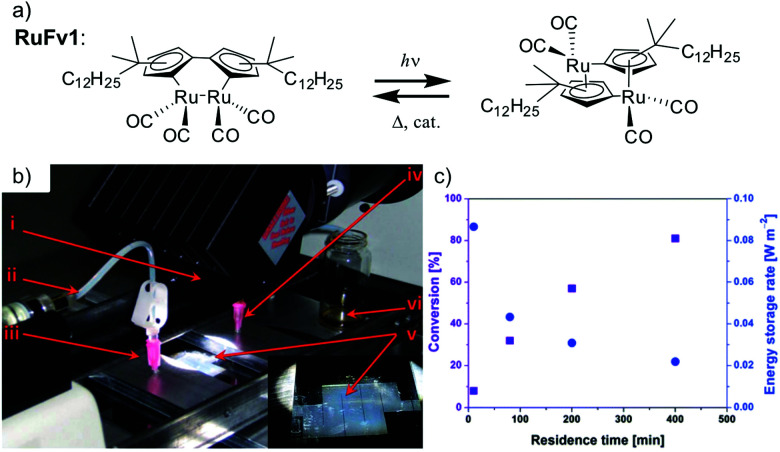
(a) Structural changes of RuFv1. (b) MOST charging device setup: (i) AM 1.5 solar simulator incorporating a 400 nm cut-off filter, (ii) syringe pump to adjust the flow rate, (iii) inlet port of solution, (iv) outlet port of solution, (v) microfluidic chip with ∼50 μm optical depth and a total surface area of 585 mm^2^, (vi) collector of irradiated solution. (c) Conversion of RuFv1 (purple squares) to its photoisomer (monitored by NMR) and energy storage characteristics (blue circles) as a function of residence time. Energy storage rate = [conversion rate (mol s^−1^) × storage enthalpy (J mol^−1^)]/area of irradiation (m^2^). Figure reproduced from ref. 24. Copyright: The Royal Society of Chemistry, 2012.

The solar collector for charging these systems can be designed in three different ways with increasing light-concentrating capacities (see Fig. 2).^21,30^ Firstly, the photoconversion device can be made in a flat panel with meander-shaped channels inside (see Fig. 2a). In laboratory experiments, this only requires a calibrated solar irradiation source with appropriate irradiance (normally with AM 1.5) on top of the panel and different fluidic residence times to accomplish the tests. Unfortunately, comparing the molecular charging performance between studies is not easy since different charging devices and molecular concentrations were used. In 2012, Segalman *et al.* used the first microfluidic chip device with ∼50 μm optical depth and a total surface area of 585 mm^2^ to convert a soluble derivative of a (fulvalene) diruthenium compound (RuFv1, see Table 1) in toluene.^24^ The diruthenium compound absorbed photons at around 450 nm and showed an energy storage density of 95 kJ mol^−1^. Unfortunately, the quantum yield was very low (0.2%), so most of the absorbed photons were wasted as molecular relaxation to heat. With a 0.1 M toluene solution, a conversion of 81% and an energy storage efficiency of 0.01% were reported (see Fig. 3).

In 2019, Mikkelsen *et al.* established a device simulation framework to predict thermal energy storage rates, conversion percentages, and temperature increases for a given MOST system.^31^ Their model was based on microscopic parameters including extinction coefficients, photoisomerization quantum yield, and energy storage density. This model has been used to simulate the conversion of a dihydroazulene derivative (DHA1, see Table 1) in a microfluidic chip of given size. The simulated results were confirmed by an experimental DHA1 conversion.^30^ This experiment was performed using a fused silica microfluidic chip with a channel depth of 85 mm, an effective exposed volume of 35.72 μL, and an irradiation surface of 5.18 cm^2^. The onset of absorption by DHA1 was 450 nm, with a *ϕ*_iso_ of 60% in toluene and a calculated energy storage density of 0.14 J g^−1^. Using 1 mM and 4 mM concentrations of DHA1 in toluene, different conversion percentages were successfully measured *via* various fluidic residence times, and a maximum energy storage efficiency of 0.1% was demonstrated with in-line flow cells and a spectrophotometer.^30^

**Fig. 4:**
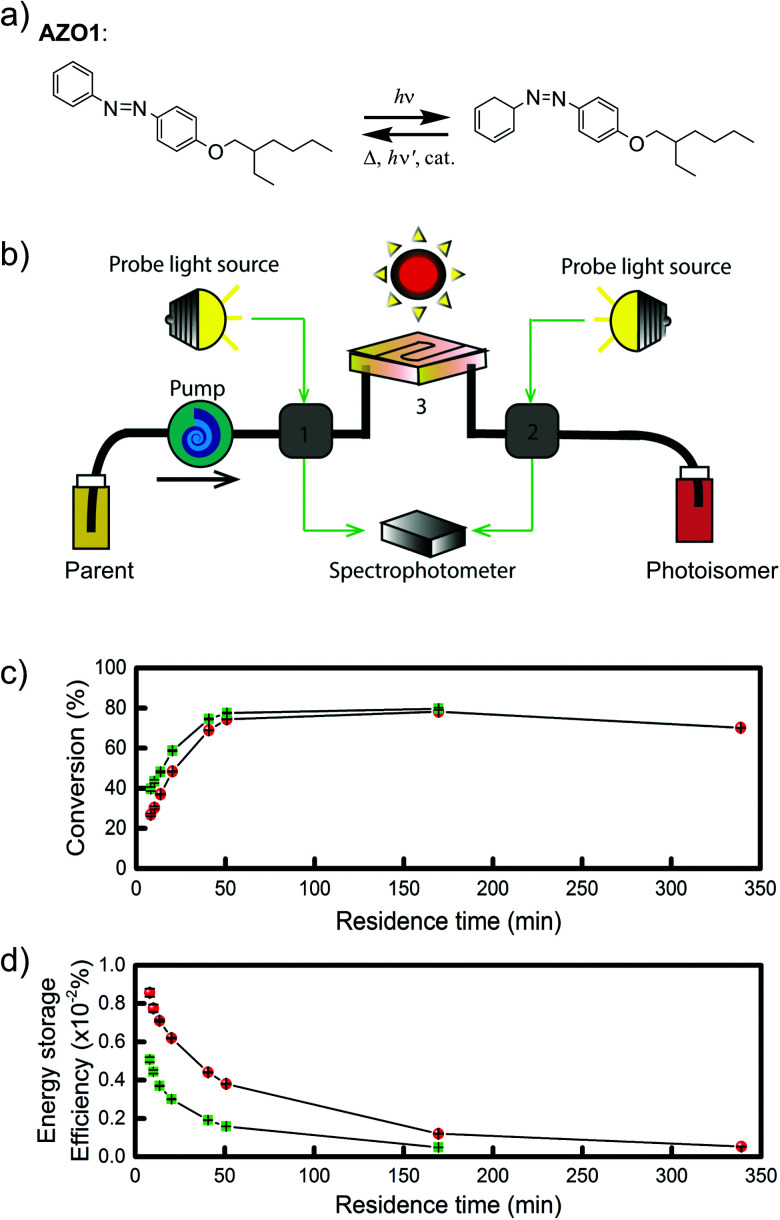
(a) Structural changes of the AZO1 derivative. (b) MOST charging device setup: gray boxes 1 and 2 are flow cells connected to UV-vis spectrophotometer; 3 is a quartz chip with 100 μm optical path length, and a total volume of 33.9 mm^3^. (c) Experimental data of the two concentrations used: 0.2 mM in green and 0.5 mM in red. Conversion percentage of AZO1-trans with different residence times in the microfluidic chip. (d) Measured energy storage efficiency of the AZO1 compound in toluene. Figure reproduced from ref. 26. Copyright: The Royal Society of Chemistry, 2019.

Later, Moth-Poulsen *et al.* designed a similar microfluidic chip solar collector with 100 μm deep channels to test the various conversion conditions of azobenzene derivatives, including AZO1 (see Table 1 and Fig. 4a).^26^AZO1 absorbed photons from 514 nm, which was higher than the absorption of the diruthenium complex. AZO1 also gave a higher photoisomerization quantum yield of 21% and a Δ*H*_storage_ of 167.5 J g^−1^. However, AZO1 is a positive photochrome and the photoisomer absorption spectrum is more red-shifted than that of its parent state, so it can also be back-converted by visible light (see Fig. 4b). Consequently, it is difficult to achieve 100% conversion. To address this, a bandpass filter was used to prevent the light-induced back-conversion from its *cis* to *trans* state. Based on these findings, negative photochromism (in which the photoisomer has a more blue-shifted onset of absorption than the parent state) is preferred for MOST applications because of the less inner filter effect. From the experimental results, even with low concentrations of 0.2 mM and 0.5 mM in toluene, the maximum conversion was around 80% in both cases, with an experimental maximal energy storage efficiency of 0.01% (see Fig. 4c and d). Recently, inspired by multijunction solar cells, a liquid-based multijunction MOST device was also experimentally demonstrated and it showed a total energy storage efficiency of 0.02% with a triple microfluidic-chip system.^16^ The overall energy storage efficiency of the whole operating device was higher than the efficiency of any of the single-layer devices, suggesting that this may be a way to improve efficiency in future devices.

**Fig. 5:**
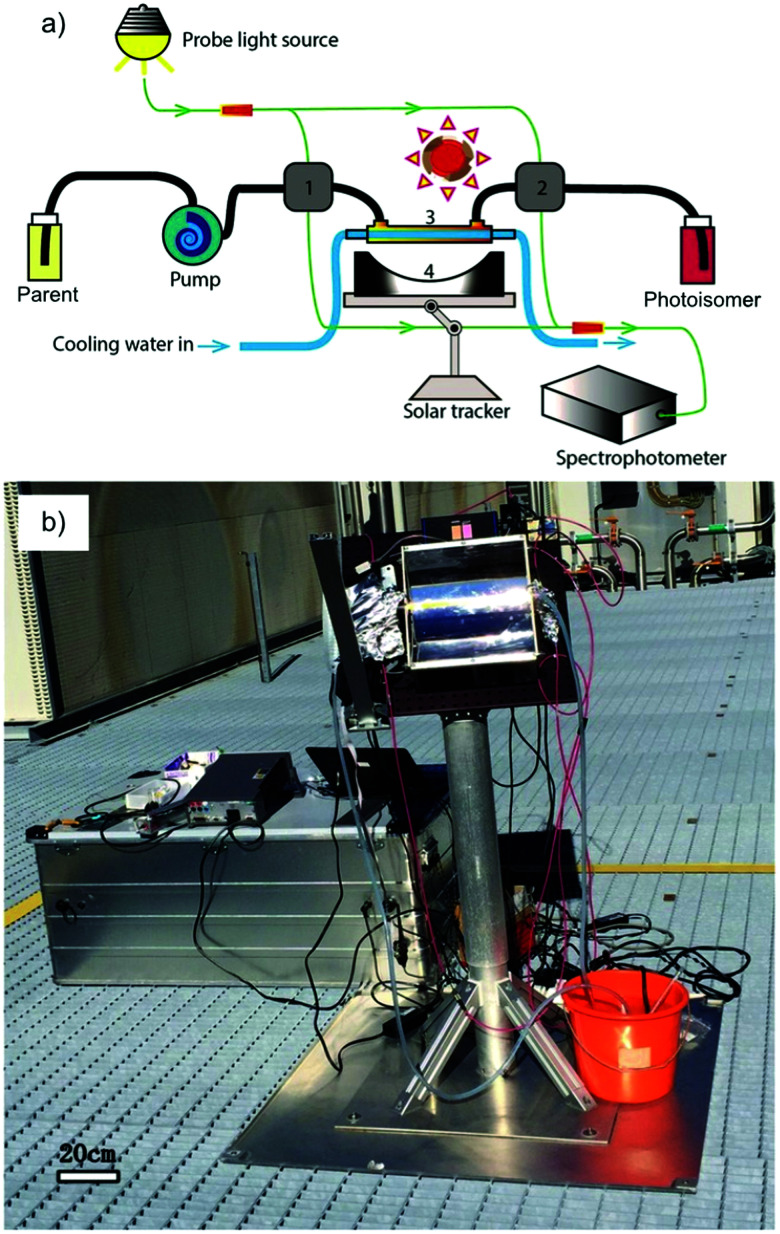
(a) Scheme of an outdoor MOST charging facility with a parabolic setup. (b) Experimental setup. Figure reproduced from ref. 30. Copyright: Wiley, 2017.

Similar to traditional reflecting solar concentrators, MOST charging devices can also be made using a parabolic reflecting mirror.^19,22,30^ For liquid MOST systems, fluidic tubing can be installed in the focus line of the parabolic collector (see Fig. 2b). In this design, the incoming photons are reflected towards the central area, where MOST liquids are pumped through.^22^ The central focusing glass tubing can be heated because of the concentrated photon flux (concentration capacity of 5–30 suns). However, this can be inconvenient for MOST applications, since the photoisomer half-life is a temperature-dependent variable.^6^ The hotter the environment is, the shorter the half-life will be. To solve this issue, the central tubing can be changed to a double-layer glass tubing; the MOST solution flows through the outer tubing and cooling water flows through the inner tubing to stabilize the system (see Fig. 5a and b). Studies have been carried out with DHA1 and NBD1 under solar irradiation in Sweden.^19,30^ In these studies, the outdoor conversion temperature was cooled to ambient temperature to maintain half-lives at the same length. With 4 mM DHA1 and 4 mM NBD1 in toluene solution, energy storage efficiencies of 0.02% and 0.03% were achieved, respectively. Later, solar collectors that can concentrate solar energy in a very limited area with a parabolic dish have also been proposed. However, the concentration ratio of these collectors can be 100–10 000 suns, which would damage the MOST molecules. Therefore, cooling would be a major issue for practical MOST applications. So far, this type of solar collector has only been incorporated in theoretical calculations as a part of conceptional devices, which will be discussed later in Section 5.^32,33^

**Fig. 6:**
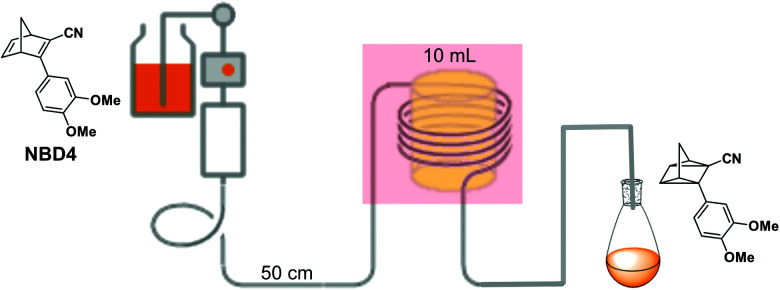
Automation flow conversion reactor (coiled tube in the photoreactor with a LED irradiation source, 365 nm) for the NBD charging process with 0.8 M in toluene-d_8_. The whole conversion process is then efficiently reduced to only 30 minutes (flow rate 0.33 mL min^−1^). Figure reproduced from ref. 21. Copyright: Elsevier, 2022.

In current converting devices, highly concentrated solutions can amplify the inner filter effects, making the conversion process extremely challenging. For instance, converting concentrated parent molecules for further energy release using the two previously mentioned setups can be time-consuming. A possible solution is to use a continuous flow conversion setup with powerful lamps as the source of photons (see Fig. 2c). In 1983, Marangoris *et al.* established a setup for the continuous conversion of unsubstituted NBD2.^34^ In this design, the NBD2 solution was pumped through a glass tube surrounded by a concentric cylindrical UV low-pressure Hg lamp. The sample was continuously charged over cycles and stabilized in a constant temperature bath. Similar to this, Yamakita *et al.* (1987) designed a small-scale continuous irradiation system. The system also continuously pumped the solution inside the loop so that the unsubstituted AZO2 (see Table 1) cyclohexane solution was charged over various cycles to enhance conversion to the *Z*-state.^35^ Recent progress in flow chemistry has led to the development of commercially available photoreactors with a high-intensity LED light source, which can simplify the conversion process. Moth-Poulsen *et al.* successfully prepared a 0.8 M NBD4 (see Table 1) toluene solution using a UV-LED irradiation source (365 nm) of 16 W and efficiently reduced the conversion process to 30 minutes (flow rate 0.33 mL min^−1^, see Fig. 6).^21^

**Fig. 7:**
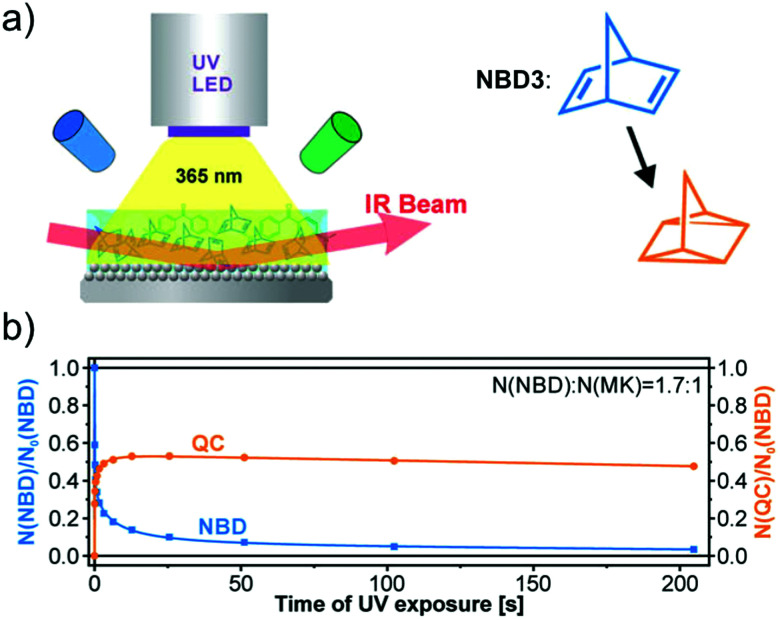
(a) Schematic representation of the experimental setup, with a UV source, a UHV chamber, and a FT-IR spectrometer. (b) Thin-film conversion experiment of NBD3 mixed with Michler's ketone (MK) in a 1.7 : 1 ratio against increasing UV exposure time. Figure reproduced from ref. 36. Copyright: AIP publishing, 2019.

In addition to devices applied for charging purposes, there are also devices for fundamental investigations. For example, Libuda *et al.* designed a high-intensity UV-photon source operating in an ultra-high vacuum environment (UHV) to study *in situ* photochemical reactions in organic thin films on a controlled surface (see Fig. 7a).^36^ The irradiation source (365 nm) was mounted in a UHV infrared reflection absorption spectroscope, allowing the photochemical process to be tracked *in situ* on thin films by infrared spectroscopy, inspiring further MOST film development. In this work, unsubstituted NBD3 (see Table 1) was converted with the help of photosensitizer 4,4-bis(dimethylamino)benzophenone (Michler's ketone). Herein, the conversion was observed for the first time in thin films (see Fig. 7b). The use of a sensitizer is an alternative way to red-shift the absorption of the MOST system and offers the opportunity to do this without having to engineer the MOST system itself.

## 4. MOST heat release devices

Normally, energy can be discharged from MOST systems in four different ways: thermal discharge, catalytic discharge, optical discharge, and electrochemical discharge.

First, one needs to consider the parameters influencing the maximum heat release temperature from a charged MOST candidate. The maximal temperature increase under adiabatic conditions in a neat sample can be calculated by the energy storage enthalpy and specific heat capacity of the photoisomer as shown in eqn (5):5
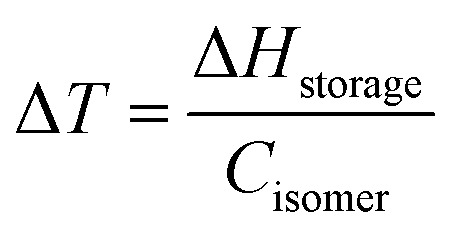


However, MOST candidates are generally in a solid state and need a solvent to become liquid for devices. Therefore, the heat capacity of the used solvent has to be taken into account:6
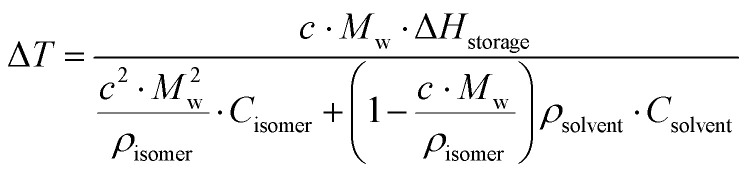


Here, *c* is the concentration of the MOST solution and *M*_w_ is the corresponding molecular weight. *C*_isomer_ refers to the specific heat capacity of the material in J g^−1^ K^−1^. *ρ*_solvent_ and *C*_solvent_ correspond to the volumetric mass density in g L^−1^ and the specific heat capacity in J g^−1^ K^−1^ of the solvent, respectively. When the concentration approaches the neat state without solvent, the 
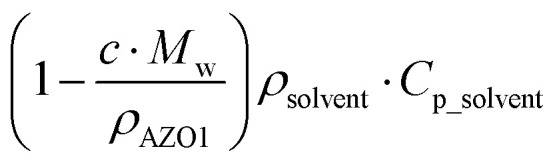
 term is zero, and the whole equation can be written as eqn (5). In practical devices, the heat capacity of the full system, as well as various heat losses, also has to be considered when assessing performance.

Of the four-heat extraction routes, a heterogeneous catalyst is the most convenient way to release energy stored in the MOST system.^11^ The catalyst needs to be fix-bedded, reusable, and independent of the MOST solution. Therefore, focus has been placed on device development with catalysts. Catalyst development for different MOST systems is crucial. The turnover number (TON) and turnover frequency (TOF) are commonly used to quantify the features of the selected candidate. The higher the TON or TOF of the catalyst, the more efficient the catalysis is. The most convenient way to design a catalyst reactor is to load the catalyst inside a tube to form a fixed bed reactor, and then flow the charged MOST solution through the reactor to release heat.

**Fig. 8:**
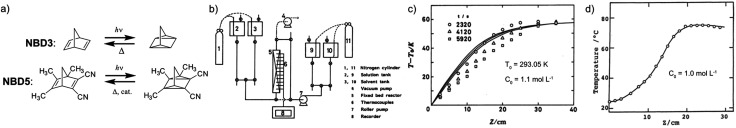
(a) Molecular structures of NBD3 and the derivative NBD5 used for macroscopic heat release experiments. (b) Experimental setup scheme. (c) Macroscopic heat release experimental results with NBD3; circle, triangle, and square represent different pumping speed. (d) Macroscopic heat release experimental results with NBD5. Figure reproduced from ref. 37 and 38. Copyright: The Chemical Society of Japan, 1988.

In 1988, Yoshida *et al.* demonstrated a catalyst setup.^37^ Using unsubstituted NBD3, cobalt(ii) deuteroporphyrin and cobalt(ii) tetrakis(*p*-sulfonatophenyl)porphin anchored on alumina beads coated with polyaminesulfone-A (Co(ii)DPIX/PAS/Alumina), they observed a maximum heat release temperature of 58.5 °C (see Fig. 8a–c). Unfortunately, unsubstituted NBD cannot operate under solar irradiation since it starts absorbing photons in the UV range at 300 nm. With a substituted NBD5 derivative (see Table 1), a temperature increase of Δ*T* = 50.5 °C was recorded in the central part of a tube-shaped fixed bed catalyst device with an inner diameter of 4 cm and a length of 58 cm (see Fig. 8a and d).^38^

**Fig. 9:**
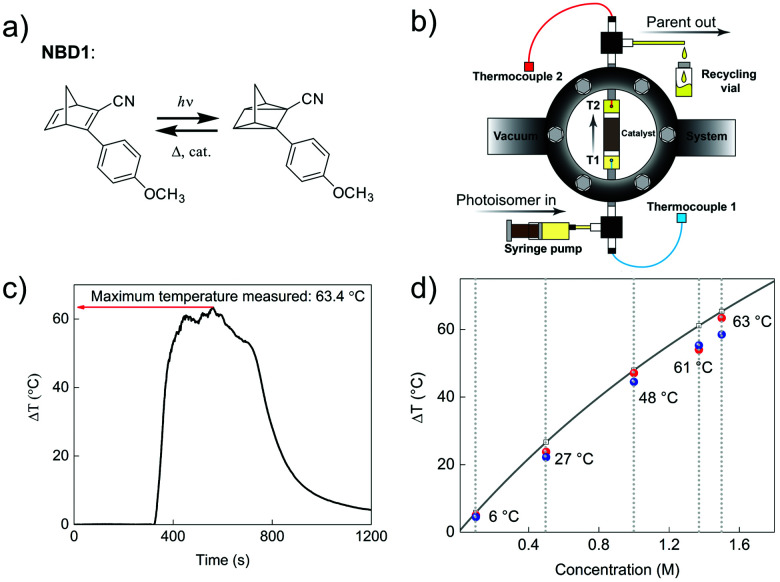
(a) Molecular structures of NBD1 used for macroscopic heat release experiments. (b) Setup of the vacuum chamber-based macroscopic heat release experiment. (c) A maximum temperature difference of 63.4 °C was recorded. (d) Macroscopic heat release with different NBD1 concentrations in toluene. Blue and red dots show the results of two experiments for each selected concentration. Figure reproduced from ref. 19. Copyright: The Royal Society of Chemistry, 2019.

In 2019, Moth-Poulsen *et al.* used cobalt phthalocyanine (CoPc) physisorbed on an activated carbon support as the catalyst for converting NBD1.^19^ Only 5 mg of CoPc@C was loaded onto Teflon tubing (1 cm long and 1 mm internal diameter). The catalysis reactor was inserted inside a vacuum chamber to prevent heat dissipating to the surrounding area (see Fig. 9a and b). Macroscopic heat release with various concentrations was measured under vacuum conditions, and the results were calculated using a simplified version of eqn (7) (see Fig. 9c). Temperatures up to 63.4 °C were captured with a catalytic TON of 482 and a TOF of 2.0 s^−1^ (see Fig. 9d). Similar experiments using the same type of catalysis reactor have also been carried out for DHA1,^30^AZO1,^26^ and RuFv1,^24^ but these experiments did not record significant heat release.

**Fig. 10:**
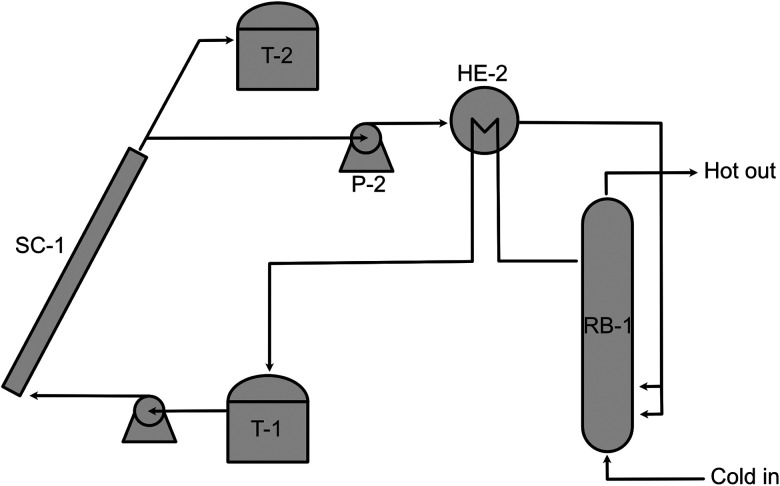
Upscaled MOST charging and heat release exchange setup. Figure adapted from ref. 34. Copyright: American Chemical Society, 1983.

Thermal discharge is the natural way to back-convert the MOST molecule. However, this might not be the most convenient way to release energy on demand. The charged photoisomer always exhibits a specific half-life of energy storage that follows the basic thermodynamic rules; the higher the temperature the molecule sustains, the faster the thermal discharge can proceed (*i.e.*, the shorter half-life the molecule will have). Not many MOST discharging devices have been designed based on thermal discharge methods because a design objective of a MOST system is a long enough half-life (*i.e.*, months or years). In 1983, Marangoris *et al.* evaluated a large-scale MOST system that was based on unsubstituted quadricyclane (QC, photoisomerized state of NBD3 in Table 1) using a thermal back-conversion energy-releasing method. The setup is shown in Fig. 10.^34^ The NBD3 solution was initially stored in a storage tank (T-1), then pumped to a flat plate solar collector (SC-1) to charge up to the QC form. The charged photoisomer solution was then stored in a second storage tank (T-2). To extract energy, the solution was then pumped by pressurized pump P-2 to heat exchanger HE1. This initiated the thermal back-conversion, allowing water to be boiled in reactor boiler RB-1 over many cycles. Although the system was technically feasible, it was not economically competitive at the time because the photoisomerization quantum yield (90% with sensitizer for unsubstituted NBD3) and the solar energy storage efficiency (0.23% for NBD3) were low. In 1984, the same research group developed a larger setup to investigate the kinetic discharge of QC.^39^ The sample was heated constantly at high temperatures (139 °C to 189 °C) and analyzed only periodically using gas chromatography.

**Fig. 11:**
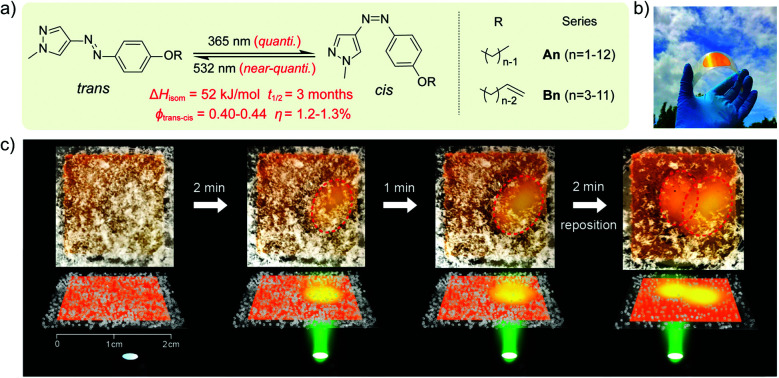
(a) Molecular structure of phase change properties of combined AZO derivatives. An and Bn represent two series with different alkyl chains. (b) Photograph of a flexible film with 5 cm diameter. (c) De-icing test using AZO derivative liquid film (1.8 cm × 1.8 cm) sandwiched between two glass slides. 532 nm light was irradiated from the back side. Figure adapted from ref. 27. Copyright: American Chemical Society, 2021.

Another way to release energy in MOST systems is by optical discharge at a specific wavelength. This method has mainly been used for azobenzene-based compounds in film samples because of the photoreactivity of the *Z*-form of the system. LEDs or lasers have been used to trigger the release of stored energy. Recently, the azopyrazole derivative AZO3 has been studied. AZO3 (see Table 1 and Fig. 11a) has combined MOST and phase change material (PCM) properties to further store and release heat in the film state (see Fig. 11b). Similar results have been reported by Li *et al.*^27^ and Han *et al.*^40–42^ Instead of domestic heating application, de-icing experiments have been successfully demonstrated by using **AZO3** (see Fig. 11c). The idea was initially proposed by Grossman *et al.* in 2013 for smart window applications, and later modeled in detail by Li *et al.* and different groups.^20,27,43–45^

The final energy-releasing route of MOST systems is electrochemical back-conversion. This technique can be an auto-catalytical process. Libuda *et al.* used Pt(111) electrodes to electrochemically trigger the back conversion of NBD2*via* the QC+• intermediate to release stored energy. Later, high-oriented pyrolytic graphite electrodes were used with a norbornadiene derivative to switch the molecule with more than 99.8% reversibility. Similar studies have also been focused on unsubstituted AZO2 and derivatives. In these studies, the back-conversion of *Z* to *E* was explained by the reductive radical cascade reaction pathway.^6^ This energy release route has the potential to release the stored energy efficiently as a catalytic method, while at the same time improving our understanding of the MOST molecular energy landscape.

## 5. MOST hybrid devices

As previously mentioned, with a multijunction design, an ideal MOST system has a maximum energy storage efficiency of up to 21% (simulated result).^16^ Therefore, it is interesting to investigate how the remaining part of the solar spectrum can be utilized. One way is to combine the MOST concept with other technologies to form a hybrid device that can increase solar energy utilization.

To use more photons from the solar spectrum, triplet–triplet annihilation (TTA) photon upconversion technology has been added to a flat-panel device. This idea has been realized with a fulvalene diruthenium derivative.^25^ The upconversion system consists of palladium(ii) octaethylporphyrin (PdOEP) as a sensitizer and 9,10-diphenylanthracene (DPA) as an annihilator. Two PdOEP compounds absorb two low-energy photons each from the visible area, and quickly relax after photoexcitation to their lowest triplet state with high quantum efficiency. The two triplet state molecules then transfer their energy to the annihilator DPA *via* Förster resonance energy transfer. After that, DPA emits a single high-energy photon, which is reabsorbed by the photoswitches for the photoisomerization reaction. The detailed process can be expressed as

7 ^1^PdOEP + *hν*_lowenergy_ → ^3^PdOEP*

8 ^3^PdOEP* + ^1^DPA → ^1^PdOEP + ^3^DPA*

9 ^3^DPA* → ^1^DPA* + *hν*_highenergy_

10 *hν*_highenergy_ + Parent → Photoisomer

**Fig. 12:**
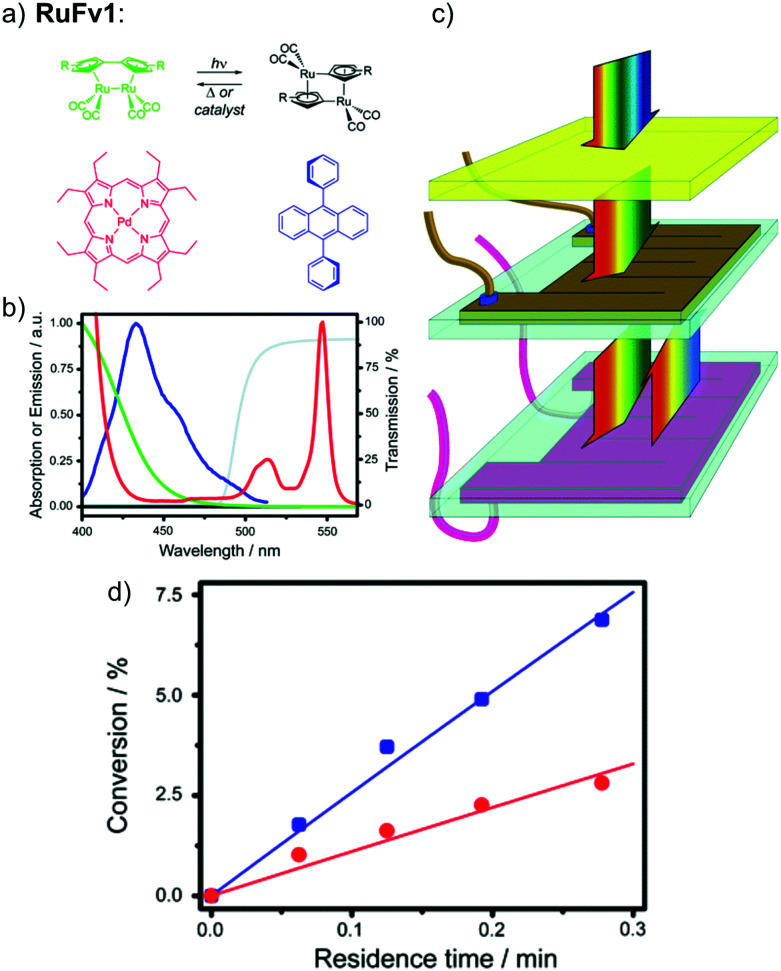
Photon upconversion facilitated molecular solar energy storage. (a) The RuFv1 derivative in toluene used in the MOST system and the TTA photon upconversion system based on the sensitizer PdOEP (red) and emitter DPA (blue). (b) Normalized absorption spectra of the ruthenium complex (green/black) and PdOEP (red), the fluorescence spectrum of DPA in the presence of PdOEP (blue), and transmission spectrum of the glass filter (grey). (c) Experimental setup. The middle chip contains the flowing MOST solution and the bottom one contains the TTA photon upconversion fluid. A glass filter blocks the absorption of PdOEP at UV wavelengths (cut-on wavelength = 495 nm). (d) Conversion efficiency (%) of photoisomerized RuFV_2_, in the presence (blue squares) and absence (red circles) of TTA photon upconversion, as a function of residence time in the microfluidic flow reactor. Figure adapted from ref. 25. Copyright: The Royal Society of Chemistry, 2013.

In this work, a photon upconversion layer was put on top of the microfluid chip device with RuFv1 solution (see Fig. 12a–c). This finally enhanced the charging of the device by 130% (see Fig. 12d). However, to amplify the observation, a bandpass filter was used to remove incoming UV light in the sensitizer absorption region.

**Fig. 13:**
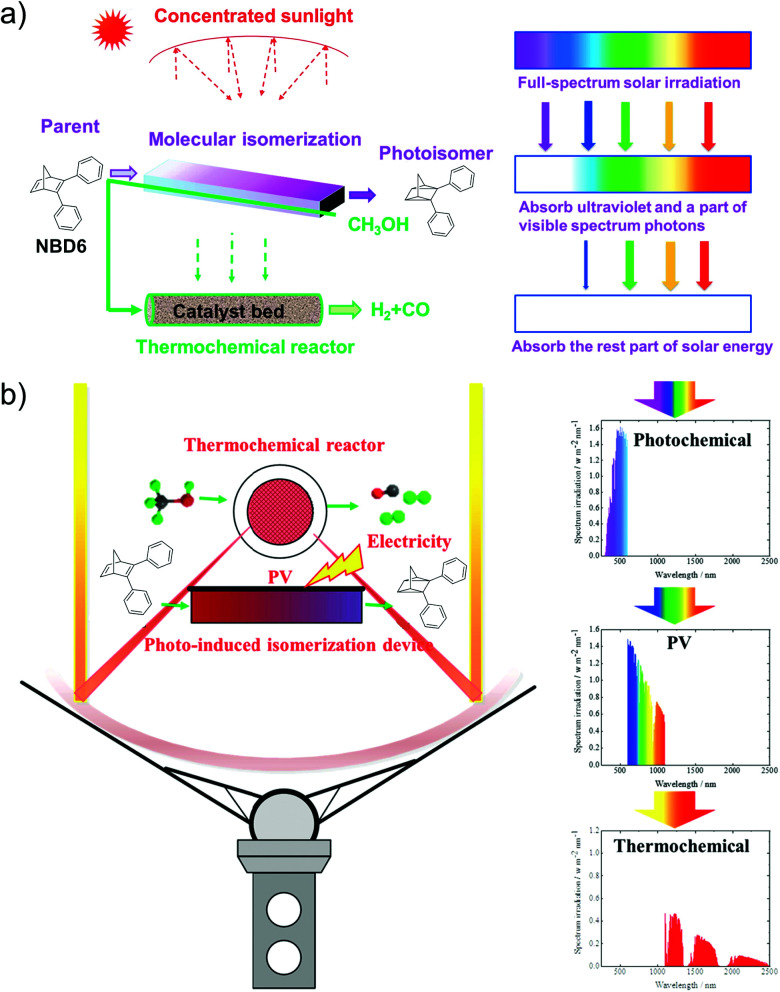
(a) Hybrid MOST solar thermochemical device and the schematic diagram of the cascade utilization of sunlight. (b) Photovoltaic-thermochemical device and the schematic diagram of the cascade utilization of sunlight. Figure reproduced from ref. 32 and 33. Copyright: Elsevier, 2019, 2020.

To utilize the full solar spectrum, Lei *et al.* suggested combining MOST with the thermochemical process. The concept was to concentrate the incoming sunlight with a parabolic solar collector on a fluidic MOST device with NBD6 solution (see Table 1).^32^ In theory, some solar energy can firstly be stored as chemical energy inside the molecules. The transmitted light can then be absorbed by a thermochemical reactor where methanol (CH_3_OH) decomposition produces hydrogen and carbon oxide gas. This hybrid system has an estimated maximum solar to chemical efficiency of 69% (see Fig. 13a). This concept was further developed by adding a photovoltaic system (see Fig. 13b).^33^ Unfortunately, this did not improve the total solar utilization efficiency. The system only attained 67% solar utilization efficiency under these conditions, where 7% of the incoming solar energy was stored in MOST and 45% was transformed into the thermal chemical process. The remaining 15% of the photons were converted into electricity. Interestingly, the estimated daily average solar utilization of this device was calculated to be 58% in summer and 45% in winter (in Hebei area, China), suggesting that this type of design can be a promising renewable energy strategy.

**Fig. 14:**
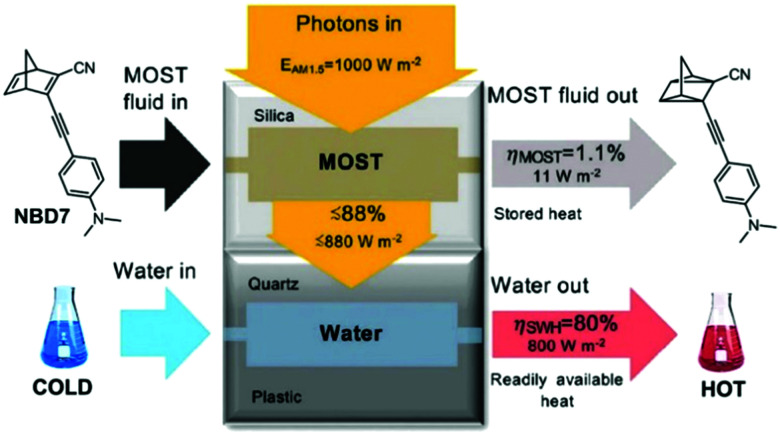
Hybrid MOST solar water heating device. The upper part is used to photoisomerize NBD7, and the bottom part is used to heat water. Up to 81% of the solar energy can be stored in the operating device. Figure reproduced from ref. 23. Copyright: The Royal Society of Chemistry, 2017.

Hybrid devices have also been tested experimentally. In 2017, Moth-Poulsen *et al.* developed a hybrid device that combined solar water heating and MOST.^23^ In this study, a fluidic fused silica-based MOST chip was set on top of a water circulating device that was 3D printed using plastic with a quartz cover. A triple-layer insulating mask composed of cardboard, insulating plastic, and aluminum foil was used to prevent unexpected heating from the solar irradiation (AM 1.5). Using NBD7 (see Table 1) with an absorption onset of 456 nm, up to 1.1% of the incoming solar energy was stored as chemical energy without affecting the solar water heating. This led to a combined solar utilization efficiency of up to 80% (see Fig. 14).

**Fig. 15:**
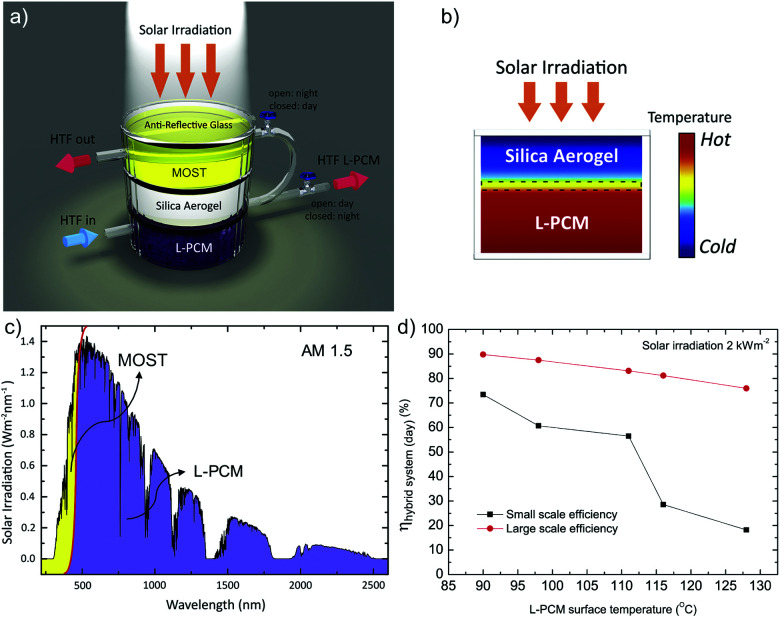
(a) MOST-PCM hybrid device setup. The silica aerogel was able to hold a constant temperature difference between the L-PCM and MOST material, which was considered a critical component for the daytime functionality of the hybrid system. (b) The L-PCM was separated by a silica aerogel to maintain the necessary temperature difference with the MOST material on top. (c) During the daytime, up to 97% of solar photons can be absorbed by the L-PCM, and the rest of the energy can be stored in the MOST material for nighttime use. (d) Energy harvesting efficiency for small-scale and large-scale tests. Figure reproduced from ref. 18. Copyright: Elsevier, 2019.

In another example, Ghasemi *et al.* coupled a MOST device with phase change materials for full solar spectrum utilization.^18^NBD1 was coupled with localized phase change materials with carbonized rayon (L-PCM, 54.1% KNO_3_, 20.0% NaNO_3_, 25.9% LiNO_3_) for potential 24/7 energy delivery (see Fig. 15a). Technically, MOST molecules and phase change materials were isolated by low thermal conductivity in a highly transparent silica aerogel to maintain an appropriate temperature difference (see Fig. 15b). The system further uses a heat transfer fluid (HTF) to harvest the heat. During the daytime, a valve that connects to the MOST layer is closed so that the MOST material could charge under solar irradiation. The valve on the bottom PCM layer is open, allowing the HTF to flow through the L-PCM to transport the released heat to be used for further heating (see Fig. 15c). During the nighttime, the upper valve is open; the charged MOST material releases the stored energy as heat, initiated by the threshold temperature from the L-PCM transferred by the HTF. This device harvested 73% of the 2 kW m^−2^ solar energy during the daytime. In a large-scale device, the two-dimensional side loss can be ignored, and the maximum harvesting efficiency can reach 90% (see Fig. 15d).

**Fig. 16:**
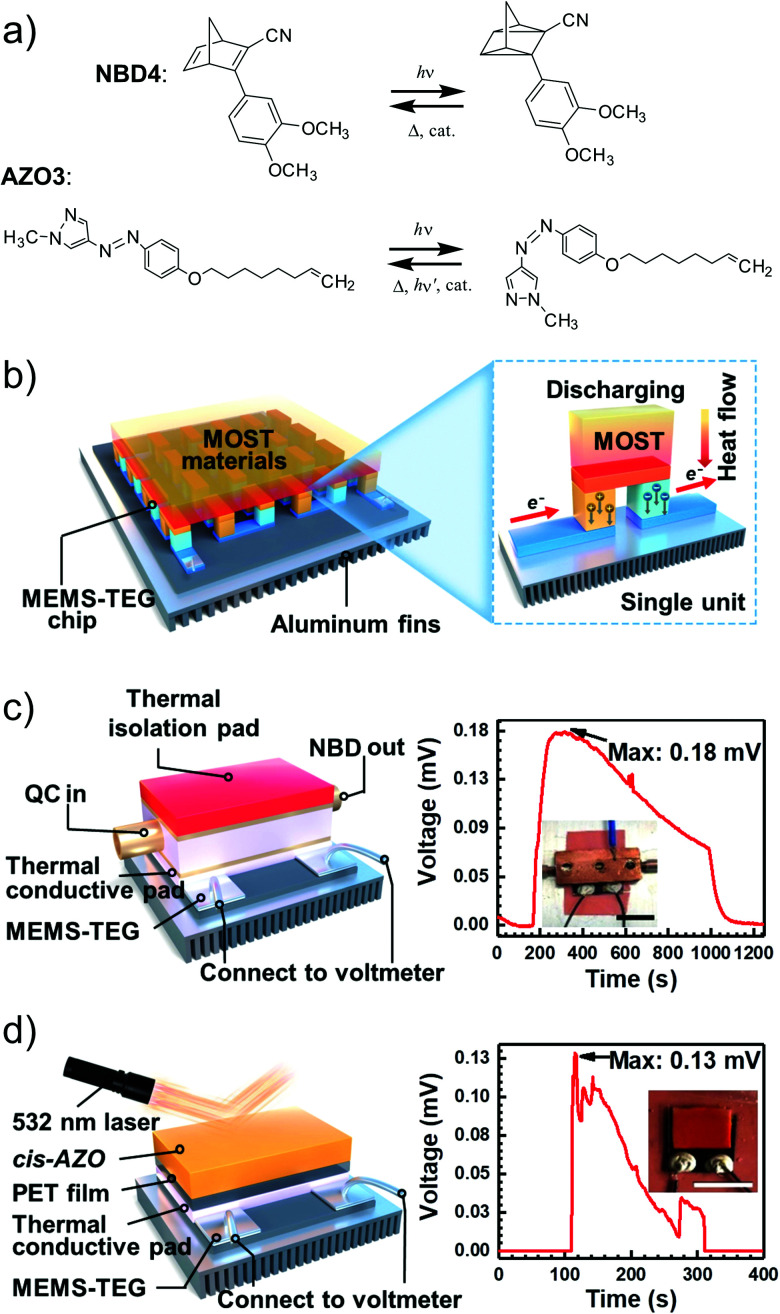
(a) MOST molecular structures used to generate power. (b) Release of stored energy for power generation. (c) NBD4 couple-based solar energy storage for the power-generation experimental setup. The net voltage generated from the MEMS-TEG chip over time. The inset is a photo of the device. Scale bar, 1 cm. (d) Schematic experimental setup of AZO3 film-based solar energy for power generation. The net voltage generated from the MEMS-TEG chip over time. The inset is a photo of the device. Scale bar, 1 cm. Figure adapted from ref. 21. Copyright: Elsevier, 2022.

MOST systems have also been combined with material-based microelectromechanical system thermoelectric generators (MEMS-TEG) to generate power. Moth-Poulsen *et al.* used an NBD derivative solution (0.78 M NBD4 in toluene, Δ*H*_isom_ = 93 kJ mol^−1^, 0.37 MJ kg^−1^) and an AZO derivative (AZO3, Δ*H*_isom_ = 52 kJ mol^−1^, 0.17 MJ kg^−1^, Δ*H*_phase_change_ = 50 kJ mol^−1^, 0.16 MJ kg^−1^, see Table 1) as a neat phase-changeable film together with an ultra-thin TEG to generate electric power up to 1.3 W m^−3^ (see Fig. 16a and b).^21^ Using both catalytic (CoPc@C fix-bedded) and optical (LED light irradiation) triggers for the back reaction, this system generated power continuously for almost 30 minutes (see Fig. 16c and d), demonstrating for the first time that MOST technology can transfer solar energy into electricity beyond time and geographical restrictions, and can thus be used as a new type of carbon-based heavy metal free solar battery.

**Fig. 17:**
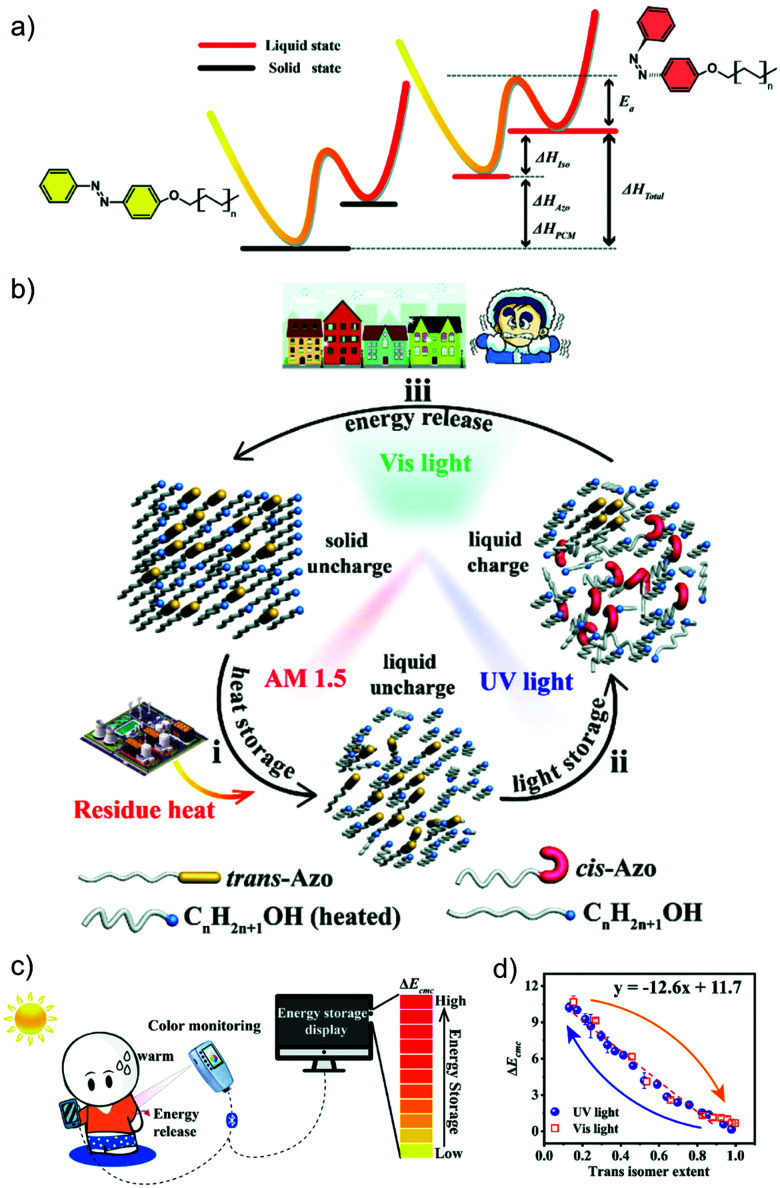
(a) Molecular structure and energy diagram of AZO phase change materials. (b) Operation principle of the AZO dopants. (c) Device working principle of the AZO phase change materials. (d) The determined correlation between color differences and isomerization extents. Figure adapted from ref. 28. Copyright: Wiley, 2020.

In recent years, more and more studies have combined MOST with other technologies, especially wearable fabrics. For example, in addition to MOST devices, color displays have also been investigated with azobenzene derivatives and phase change materials. Wang *et al.* presented a series of photochromic azobenzene dopants on PCMs with different molar ratios. The energy density of the system reached 240 J g^−1^ (Fig. 17a and b)^28^ and more than 6 °C of released heat was captured by the thermal camera. Meanwhile, since AZO derivatives change color during charging, a correlation was determined between conversion percentage and color change. This has high potential to be developed into a visual solar thermal energy system (Fig. 17c and d).

**Fig. 18:**
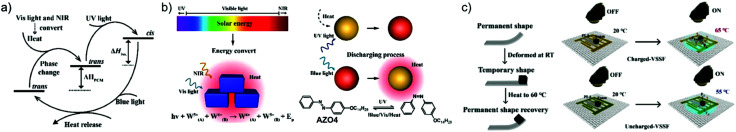
(a) Energy diagram of the AZO derivative with phase change property encapsulated in polystyrene. (b) Scheme showing the functioning principle of the system. (c) Photothermally driven lifter device operating principle. Figure reproduced from ref. 29. Copyright: Elsevier, 2021.

The same research group later designed a wearable MOST fabric that utilized the full solar spectrum, including visible and NIR photons.^29^ An AZO derivative, similar to AZO1 in Table 1, with phase change properties was encapsulated in polystyrene. This material effectively prevented leakage and volume change during phase transition of the AZO derivative. The nanocomposites were dispersed with Cs_0.32_WO_3_ nanoparticles and applied to the fabric surface. Such Cs_0.32_WO_3_ nanoparticles can absorb NIR light, thus transferring heat to AZO units, and then initiate the phase change property. Meanwhile, UV photons from the solar spectrum also contributed to the charging process. For discharge, blue photons from solar irradiation released the stored energy in azobenzene-PCM (Fig. 18a and b). It was determined that the total energy density for this system was 28.5 J g^−1^ (101.6 kJ mol^−1^), giving a total solar energy conversion efficiency of 5%, and an experimental heat release of 13 °C. Regarding the robustness of such a system, the charging and discharging process was repeated over 50 cycles, showing high durability of the material. Interestingly, an additional photothermally driven lifter device has also been developed (Fig. 18c). A similar idea using a nanoconfinement approach to improve energy density was demonstrated by Cai *et al.*, they used microphase separation of a block copolymer to selectively disperse phase change materials into nanodomains of a polymer film, forming new MOST composite materials.^46^ This promising method can be used to develop wearable warm fabrics. For example, the charged composite can release energy with a 460 nm visible light source, giving a maximum temperature increase of 11 °C. Since this heat increase is less than 50 °C, it will not harm human skin for further wearable application design. Besides, it was suggested that such a composite can also be combined with a polyurethane sponge to actuate the water surface under pneumatic electric field changes.

## 6. Summary, challenges, and future work

The estimated maximum MOST energy storage efficiency (20.5%)^16^ is certainly better than that of photosynthesis (0.1–0.3%).^47^ However, MOST systems must be further developed to meet the conversion efficiency of recent solar cells (up to 47.1% with a six-junction flat-plate terrestrial design under 143 suns).^48^ Based on the operation principle of the MOST concept, in which light energy is both converted and stored within the molecule without an external energy storage unit, these future devices will be constructed as a heavy metal-free and highly robust system. Unfortunately, if one considers the publication from Thanh Xuan *et al.* as the first MOST lab-to-site transfer proposal in 1979,^10^ no large-scale device has been implemented within the last 40 years. This may be because this area is still in the fundamental phase of basic molecular motif scanning; the performance of an ideal MOST device will depend on the performance of the selected molecular candidate. Further developments in synthetic, physical, and organic chemistry might uncover more red-shifted MOST molecules that have less spectral overlap with their corresponding photoisomers. Based on the economic evaluation by Marangozls *et al.* in 1983, cycling capacity is an important parameter to consider when developing MOST systems.^34^ So far, the main focus on cyclability is still at the molecular property level,^6^ and the only device cycling test was accomplished with NBD4 and a fixed-bed catalyst system.^21^ Future small- and large-scale cycling tests are certainly needed. Fortunately, improving technology means the cost of large-scale industrial implementation can largely be balanced by the energy saved. In addition, molecular screening by using machine learning and artificial intelligence (AI) technology^49^ will also accelerate the discovery of new candidates, and automation of chemistry systems^50^ will increase the potential for scaling up. However, unique and niche, small-scale applications, such as wearable MOST cloth or hybrid devices, may be areas where the MOST systems can be applied in a not too distant future.

## Conflicts of interest

There are no conflicts to declare.

## Supplementary Material

## References

[cit1] Bureau P. R. , The 2020 World Population Data Sheet, 2020

[cit2] El-Kholy M. K. T. A. , The World Environment 1972–1992, Chapman & Hall, 1992

[cit3] IEA, *Key World Energy Statistics 2021*, Paris, 2021

[cit4] Breeze P. , in Power Generation Technologies, ed. P. Breeze, Newnes, 3rd edn, 2019

[cit5] Miljanić O. Š. and Pratt J. A., Introduction to Energy and Sustainability, Weinheim, Germany, 2021

[cit6] Wang Z., Erhart P., Li T., Zhang Z.-Y., Sampedro D., Hu Z., Wegner H. A., Brummel O., Libuda J., Nielsen M. B., Moth-Poulsen K. (2021). Joule.

[cit7] Orrego-Hernández J., Dreos A., Moth-Poulsen K. (2020). Acc. Chem. Res..

[cit8] Dong L., Feng Y., Wang L., Feng W. (2018). Chem. Soc. Rev..

[cit9] Weigert F. (1909). Ber. Dtsch. Chem. Ges..

[cit10] Jones G., Chiang S.-H., Xuan P. T. (1979). J. Photochem. Photobiol..

[cit11] Yoshida Z.-i (1985). J. Photochem. Photobiol..

[cit12] Bren V. A., Dubonosov A. D., Minkin V. I., Chernoivanov V. A. (1991). Russ. Chem. Rev..

[cit13] Sun C.-L., Wang C., Boulatov R. (2019). ChemPhotoChem.

[cit14] Lennartson A., Roffey A., Moth-Poulsen K. (2015). Tetrahedron Lett..

[cit15] Börjesson K., Lennartson A., Moth-Poulsen K. (2013). ACS Sustainable Chem. Eng..

[cit16] Wang Z., Moïse H., Cacciarini M., Nielsen M. B., Morikawa M.-A., Kimizuka N., Moth-Poulsen K. (2021). Adv. Sci..

[cit17] Strubbe D. A., Grossman J. C., Condens J. (2018). Matter Phys..

[cit18] Kashyap V., Sakunkaewkasem S., Jafari P., Nazari M., Eslami B., Nazifi S., Irajizad P., Marquez M. D., Lee T. R., Ghasemi H. (2019). Joule.

[cit19] Wang Z., Roffey A., Losantos R., Lennartson A., Jevric M., Petersen A. U., Quant M., Dreos A., Wen X., Sampedro D., Börjesson K., Moth-Poulsen K. (2019). Energy Environ. Sci..

[cit20] Petersen A. U., Hofmann A. I., Fillols M., Mansø M., Jevric M., Wang Z., Sumby C. J., Müller C., Moth-Poulsen K. (2019). Adv. Sci..

[cit21] Wang Z., Wu Z., Hu Z., Orrego-Hernández J., Mu E., Zhang Z.-Y., Jevric M., Liu Y., Fu X., Wang F., Li T., Moth-Poulsen K. (2022). Cell Rep. Phys. Sci..

[cit22] Gray V., Lennartson A., Ratanalert P., Börjesson K., Moth-Poulsen K. (2014). Chem. Commun..

[cit23] Dreos A., Börjesson K., Wang Z., Roffey A., Norwood Z., Kushnir D., Moth-Poulsen K. (2017). Energy Environ. Sci..

[cit24] Moth-Poulsen K., Ćoso D., Börjesson K., Vinokurov N., Meier S. K., Majumdar A., Vollhardt K. P. C., Segalman R. A. (2012). Energy Environ. Sci..

[cit25] Börjesson K., Dzebo D., Albinsson B., Moth-Poulsen K. (2013). J. Mater. Chem. A.

[cit26] Wang Z., Losantos R., Sampedro D., Morikawa M.-A., Börjesson K., Kimizuka N., Moth-Poulsen K. (2019). J. Mater. Chem. A.

[cit27] Zhang Z.-Y., He Y., Wang Z., Xu J., Xie M., Tao P., Ji D., Moth-Poulsen K., Li T. (2020). J. Am. Chem. Soc..

[cit28] Fei L., Yin Y., Zhang J., Wang C. (2020). Sol. RRL.

[cit29] Fei L., Yin Y., Yang M., Zhang S., Wang C. (2021). Energy Storage Mater..

[cit30] Wang Z., Udmark J., Börjesson K., Rodrigues R., Roffey A., Abrahamsson M., Nielsen M. B., Moth-Poulsen K. (2017). ChemSusChem.

[cit31] Hansen M. H., Olsen S. T., Sylvester-Hvid K. O., Mikkelsen K. V. (2019). Chem. Phys..

[cit32] Fang J., Liu Q., Guo S., Lei J., Jin H. (2019). Appl. Energy.

[cit33] Fang J., Wu H., Liu T., Zheng Z., Lei J., Liu Q., Jin H. (2020). Appl. Energy.

[cit34] Philippopoulos C., Economou D., Economou C., Marangozis J. (1983). Ind. Eng. Chem. Res..

[cit35] Taoda H., Hayakawa K., Kawase K., Yamakita H. (1987). J. Chem. Eng. Jpn..

[cit36] Schwarz M., Schuschke C., Silva T. N., Mohr S., Waidhas F., Brummel O., Libuda J. (2019). Rev. Sci. Instrum..

[cit37] Sadao M., Yoshinobu A., Masayoshi M., Toshinobu O., Zen-ichi Y., Toshiro M., Masahiro F., Tomoaki T. (1988). Bull. Chem. Soc. Jpn..

[cit38] Miki S., Maruyama T., Ohno T., Tohma T., Toyama S.-I., Yoshida Z. I. (1988). Chem. Lett..

[cit39] Philippopoulos C., Marangozis J. (1984). Ind. Eng. Chem. Res..

[cit40] Qiu Q., Shi Y., Han G. G. D. (2021). J. Mater. Chem. C.

[cit41] Shi Y., Gerkman M. A., Qiu Q., Zhang S., Han G. G. D. (2021). J. Mater. Chem. A.

[cit42] Gerkman M. A., Han G. G. D. (2020). Joule.

[cit43] Shames S. W. L., Zhang C. M., Ferralis N., Grossman J. C. (2013). Int. J. Energy Efficient Veh. Des..

[cit44] Zhitomirsky D., Cho E., Grossman J. C. (2016). Adv. Energy Mater..

[cit45] Saydjari A. K., Weis P., Wu S. (2017). Adv. Energy Mater..

[cit46] Cai F., Song T., Yang B., Lv X., Zhang L., Yu H. (2021). Chem. Mater..

[cit47] Scharf H.-D., Fleischhauer J., Leismann H., Ressler I., Schleker W.-G., Weitz R. (1979). Angew. Chem., Int. Ed. Engl..

[cit48] Geisz J. F., France R. M., Schulte K. L., Steiner M. A., Norman A. G., Guthrey H. L., Young M. R., Song T., Moriarty T. (2020). Nat. Energy.

[cit49] Ree N., Koerstz M., Mikkelsen K. V., Jensen J. H. (2021). J. Chem. Phys..

[cit50] Orrego-Hernández J., Hölzel H., Quant M., Wang Z., Moth-Poulsen K. (2021). Eur. J. Org. Chem..

